# Complete genome sequences of *Aerococcus loyolae* ATCC TSD-300^T^, *Aerococcus mictus* ATCC TSD-301^T^, and *Aerococcus tenax* ATCC TSD-302^T^

**DOI:** 10.1128/mra.00156-24

**Published:** 2024-04-23

**Authors:** Brian I. Choi, Melline Fontes Noronha, Jacob Kaindl, Alan J. Wolfe

**Affiliations:** 1 Department of Microbiology and Immunology, Loyola University Chicago, Maywood, Illinois, USA; Department of Biology, Queens College, Queens, New York, USA

**Keywords:** *Aerococcus*, DNA sequencing, genomes, urobiome, human microbiome

## Abstract

Previously identified under the single designation of *Aerococcus urinae*, three distinct taxonomic species have been distinguished as *Aerococcus loyolae, Aerococcus mictus,* and *Aerococcus tenax*. Here, we present the complete genome sequences of the type strains of these species assembled via a combination of short-read and long-read sequencing techniques.

Registered at ClinicalTrials.gov (NCT01166438).

## ANNOUNCEMENT

*Aerococcus urinae* has been reorganized into four distinct species: *Aerococcus urinae*, *Aerococcus loyolae*, *Aerococcus mictus*, and *Aerococcus tenax* (1). To elucidate the genes behind pathogenic mechanisms, the complete genome sequences of the type strains are necessary. We present the complete genome sequences of the type strains of *A. loyolae* ATCC TSD-300^T^ (=UMB0080^T^ =DSM 115698^T^ =CCUG 76533^T^ = NR-58628^T^), *A. mictus* ATCC TSD-301^T^ (=UMB3440^T^ =DSM 115699^T^ =CCUG 76532^T^ = NR-58629^T^), and *A. tenax* ATCC TSD-302^T^ (=UMB3669^T^ =DSM 115700^T^ =CCUG 76531^T^ =NR-58630^T^). The strains were all originally identified as *A. urinae* via matrix-assisted laser desorption/ionization-time-of-flight (MALDI-TOF) mass spectrometry, as previously described (2). This involved plating urine on sheep blood agar plates and incubating them for 48 hours at 37°C with 5% supplemented CO_2_ gas. Single colonies were isolated and analyzed via MALDI-TOF before being cryopreserved in Brucella Broth Media with 15% glycerol.

All three type strains were isolated from urine obtained via transurethral catheter from female urogynecology patients at Loyola University Medical Center, Maywood, IL, USA. ATCC TSD-301^T^and ATCC TSD-302^T^ came from patients diagnosed with urge urinary incontinence, and ATCC TSD-300^T^ was isolated from a patient diagnosed with overactive bladder syndrome. Patients were part of a clinical trial observing urge incontinence, Study Registration No. NCT01166438 and IRB LU215192 (3, 4).

DNA extraction for short-read Illumina sequences of ATCC TSD-300^T^and ATCC TSD-301^T^ was conducted without a kit, as previously described (5). Briefly, cells were pelleted after 48-hour growth before being suspended in DNA extraction buffer (20 mM Tris-Cl, 2 mM EDTA, 1.2% Triton X-100, pH 8) and lysed with the addition of lysozyme, mutanolysin, and proteinase K. Phenol-chloroform extraction of DNA then followed. Sequencing for these two strains was conducted using the Nextera XT DNA Library Preparation Kit (Illumina) and the MiSeq Reagent Kit v2 run on the Illumina HiSeq 2000 platform for ATCC TSD-300^T^ and the Illumina MiSeq platform for ATCC TSD-301^T^. DNA extraction and short-read Illumina sequencing of ATCC TSD-302^T^ were performed by SeqCenter using the ZymoBIOMICS DNA kit and the Nextera DNA Flex Library Prep Kit (Illumina) run on the Illumina MiSeq platform. Extraction and sequencing of long-read Oxford Nanopore sequences of all strains were performed by SeqCenter using ZymoBIOMICS DNA Miniprep Kit and Oxford Nanopore Technology Ligation Sequencing kit V14 with the NEBNext Companion Module according to the manufacturer’s specifications. No additional DNA fragmentation or size selection was performed. Sequencing was conducted on R10.4.1 flowcells run on the MinION platform. Guppy v6.4.6 (Nanopore) was used for basecalling, demultiplexing, and adapter removal.

For hybrid assembly, default parameters were used for all software. Cutadapt v3.7 (6) and NanoFilt v2.8.0 (7) were used to filter low-quality sequences on raw reads, respectively. High-quality reads were assembled using Flye v.2.9 (8), SPAdes v3.15.4 (9), and Canu v.1.5 (10) for ATCC TSD-300^T^, ATCC TSD-301^T^, and ATCC TSD-302^T^, respectively. Illumina reads were used to polish long-read assemblies using Pilon v1.24 (11). Assembly qualities were validated with QUAST v5.2.0 (12) and circularized using Circlator v1.5.5 (13). Genome annotations were conducted via PGAP v6.6 (14) (Table 1).

**TABLE 1:** Complete genome characteristics

Characteristic	*A. loyolaeATCC TSD-300^T^*	*A. mictus* ATCC TSD-301^T^	*A. tenax* ATCC TSD-302^T^
Illumina sequencing			
Read length (nucleotides)	100	250	150
No. of read pairs	1,707,844	775,786	1,520,375
Nanopore sequencing			
No. of reads	752,306	381,666	1,491,021
Read *N*_50_ (bp)	652	3,039	3,434
Genome properties			
No. of contigs	1	2	2
Structure	One chromosome	One chromosome and one plasmid	One chromosome and one plasmid
Genome length (bp), chromosome (plasmid)	2,034,560	2,117,296 (7,768)	2,087,453 (9,244)
GC content (%), chromosome (plasmid)	42	42 (30)	42 (30)
Average coverage (×)			
Illumina reads	166	172	217
Nanopore reads	344	288	1,387
Annotation statistics			
Predicted no. of coding DNA sequences	1,754	1,855	1,834
No. of rRNAs (5S, 16S, 23S)	4, 4, 4	4, 4, 4	4, 4, 4
No. of tRNAs	60	60	60
GenBank accession no.	GCA_002871915.2	GCA_003286595.3	GCA_003286645.3

## Data Availability

The Illumina raw reads/Nanopore raw reads/assemblies have been deposited in GenBank under the following accession numbers: A. loyolae ATCC TSD-300T: SRR5946029/SRR25068130/GCA_002871915.2, A. mictus ATCC TSD-301T: SRR6973127/SRR25068145/GCA_003286595.3, and A. tenax ATCC TSD-302T: SRR6973128/SRR25068144/GCA_003286645.3.
